# Contest between Mr. Fergusson and Mr. Syme

**Published:** 1857-04

**Authors:** 


### *Contest between Mr. Fergusson and Mr. Syme.*

Now that the smoke of the contest, condncted, on the one hand, by Mr. Fergusson, and on the other by the equally distinguished Mr. Syme, in regard to the manner in which the late Mr. Liston was in the habit of holding the knife in the operation of lithotomy, has passed away, it turns out as we predicted, that the Scotch professor has gained the day, and the victorious party are having a gay time over the defeat of the Queen's surgeon. The last and decisive charge was made by Mr. Pirrie, whose array of facts in proof that Mr. Liston held the scalpel in the underhand position, as represented in the various wood-cuts which appear in the pages of his own, Mr. Pirrie's, Mr. Miller's, and Mr. Druitt's books on surgery, is perfectly overwhelming.

But hardly is this *great* battle of the knives decided before another equally honorable to the distinguished parties involved is begun. Mr. Ramsbotham, who, however, notwithstanding his high position in the obstetric world and the adaptative nature of his calling to the annually recurring professional wants of her Majesty, has not we believe, the entrée of the royal chamber, charges publicly upon Mr. Churchill, a brother accoucheur, a theft committed some twelve or fifteen years ago. The articles said to be stolen are certain wood-cut illustrations, which, without leave or license, or even an acknowledgment, Mr. Churchill was wicked enough to copy from Mr. Ramsbotham's book on midwifery, published about that time. The war has just begun, and how it will end the world will no doubt in due time be informed. In the meantime, speculation is rife, and the intensest interest is felt to know the result.—*North American Med. Chirurg. Review.*

